# Letter to the Editor regarding “Importance of over‐reading ambulatory ECG‐based microvolt T‐wave alternans to eliminate three main sources of measurement error”

**DOI:** 10.1111/anec.12716

**Published:** 2019-10-03

**Authors:** Raja J. Selvaraj

**Affiliations:** ^1^ Jawaharlal Institute of Postgraduate Medical Education and Research Puducherry India

Dear Editor,

In the study by Takasugi et al. (2019), the authors visually inspected episodes of T‐wave alternans (TWA) detected by the modified moving average (MMA) algorithm in ambulatory recordings from healthy persons. They identified episodes of false TWA detection due to artifacts, positional, and post extrasystolic T‐wave changes. Based on this, they advocate visual over‐reading of automatically detected TWA episodes.

With simulated waveforms, which is a better method to study the effect of artifact on TWA measurement, we have previously shown that MMA method is sensitive to noise and artifacts which can produce false positives, in contrast to the spectral method, where it results in false negatives (Selvaraj & Chauhan, 2009). Similarly, we also postulated that periodic T‐wave changes which can occur due to respiration can produce false positives when the frequency of this periodicity is 0.25. These findings have been confirmed in this study.

Signal processing and algorithms were designed to detect TWA that occurs at amplitudes not visible to the naked eye. Relying on visual over‐reading is therefore not a viable method as small amplitude T‐wave alternans would be rejected in the presence of a more visible artifact. Visual over‐reading would also be time consuming and labor intensive.

To overcome the problem of false positives due to noise, we suggested the use of a MMA ratio (Selvaraj & Chauhan, 2009), using MMA detected alternans in a series of odd or even beats only as a surrogate measure of noise. This can be automatically computed and in our simulations provides good discrimination of true versus artifactual alternans. Such an algorithmic approach, instead of visual over‐reading should be the preferred method of identifying false positive TWA.

## References

[anec12716-bib-0001] Selvaraj, R. J. , & Chauhan, V. S. (2009). Effect of noise on T‐wave alternans measurement in ambulatory ECGs using modified moving average versus spectral method. Pacing and Clinical Electrophysiology, 32, 632–641. 19422585 10.1111/j.1540-8159.2009.02337.x

[anec12716-bib-0002] Takasugi, N. , Matsuno, H. , Takasugi, M. , Shinoda, K. , Watanabe, T. , Ito, H. , … Verrier, R. L. (2019). Importance of over‐reading ambulatory ECG‐based microvolt T‐wave alternans to eliminate three main sources of measurement error. Annals of Noninvasive Electrocardiology, 26, e12670 10.1111/anec.12670 10.1111/anec.12670 PMC6931797 31241245

